# GSK3beta-Mediated Drp1 Phosphorylation Induced Elongated Mitochondrial Morphology against Oxidative Stress

**DOI:** 10.1371/journal.pone.0049112

**Published:** 2012-11-20

**Authors:** Chia-Hua Chou, Ching-Chih Lin, Ming-Chang Yang, Chih-Chang Wei, Huei-De Liao, Run-Chin Lin, Wen-Yu Tu, Tsung-Chieh Kao, Ching-Mei Hsu, Jiin-Tsuey Cheng, An-Kuo Chou, Chu-I Lee, Joon-Khim Loh, Shen-Long Howng, Yi-Ren Hong

**Affiliations:** 1 Department of Biochemistry, Faculty of Medicine, College of Medicine, Kaohsiung Medical University, Kaohsiung, Taiwan, R.O.C.; 2 Department of Biological Sciences, National Sun Yat-Sen University, Kaohsiung, Taiwan, R.O.C.; 3 Laboratory of Genetic Research, Kaohsiung Armed Forces General Hospital, Kaohsiung, Taiwan, R.O.C.; 4 Graduate Institute of Medicine, Kaohsiung Medical University, Kaohsiung, Taiwan, R.O.C.; 5 Department of Anesthesiology, Kaohsiung Chang Gung Memorial Hospital and Chang-Gung University College of Medicine, Kaohsiung, Taiwan, R.O.C.; 6 Department of Medical Laboratory Science and Biotechnology, Fooyin University, Kaohsiung, Taiwan, R.O.C.; 7 Department of Neurosurgery, Kaohsiung Medical University Hospital, Kaohsiung, Taiwan, R.O.C.; Roswell Park Cancer Institute, United States of America

## Abstract

Multiple phosphorylation sites of Drp1 have been characterized for their functional importance. However, the functional consequence of GSK3beta-mediated phosphorylation of Drp1 remains unclear. In this report, we pinpointed 11 Serine/Threonine sites spanning from residue 634∼736 of the GED domain and robustly confirmed Drp1 Ser693 as a novel GSK3beta phosphorylation site. Our results suggest that GSK3beta-mediated phosphorylation at Ser693 does cause a dramatic decrease of GTPase activity; in contrast, GSK3beta-mediated phosphorylation at Ser693 appears not to affect Drp1 inter-/intra-molecular interactions. After identifying Ser693 as a GSK3beta phosphorylation site, we also determined that K679 is crucial for GSK3beta-binding, which strongly suggests that Drp1 is a novel substrate for GSK3beta. Thereafter, we found that overexpressed S693D, but not S693A mutant, caused an elongated mitochondrial morphology which is similar to that of K38A, S637D and K679A mutants. Interestedly, using H89 and LiCl to inhibit PKA and GSK3beta signaling, respectively, it appears that a portion of the elongated mitochondria switched to a fragmented phenotype. In investigating the biofunctionality of phosphorylation sites within the GED domain, cells overexpressing Drp1 S693D and S637D, but not S693A, showed an acquired resistance to H_2_O_2_-induced mitochondrial fragmentation and ensuing apoptosis, which affected cytochrome c, capase-3, -7, and PARP, but not LC3B, Atg-5, Beclin-1 and Bcl2 expressions. These results also showed that the S693D group is more effective in protecting both non-neuronal and neuronal cells from apoptotic death than the S637D group. Altogether, our data suggest that GSK3beta-mediated phosphorylation at Ser693 of Drp1 may be associated with mitochondrial elongation via down-regulating apoptosis, but not autophagy upon H_2_O_2_ insult.

## Introduction

Glycogen synthase kinase-3 (GSK-3) is a serine/threonine kinase originally found to inactivate the enzyme glycogen synthase by phosphorylation [1]. It is well documented that GSK-3 is crucial for cell development, metabolic homeostasis, neuronal growth and differentiation, cell polarity, cell fate and apoptosis [2]. Two mammalian GSK3 isoforms, GSK3α and GSK3beta, that share 97% amino acid identity in their catalytic domain were cloned [3]. GSK3beta has attracted significant attention, in part due to its multifaceted roles in multiple key pathophysiological pathways involved in Alzheimer's disease (AD) and several neurodegenerative diseases [4]. We previously explored GSK3beta and its interacting proteins, HdynIV (since renamed dynamin related protein 1, Drp1) [5]. Functional characterization showed that this Drp1 variant lacks a proline-rich domain on its carboxyl-terminus, which was identified as a critical region for interacting with GSK3 [6].

**Figure 1:**
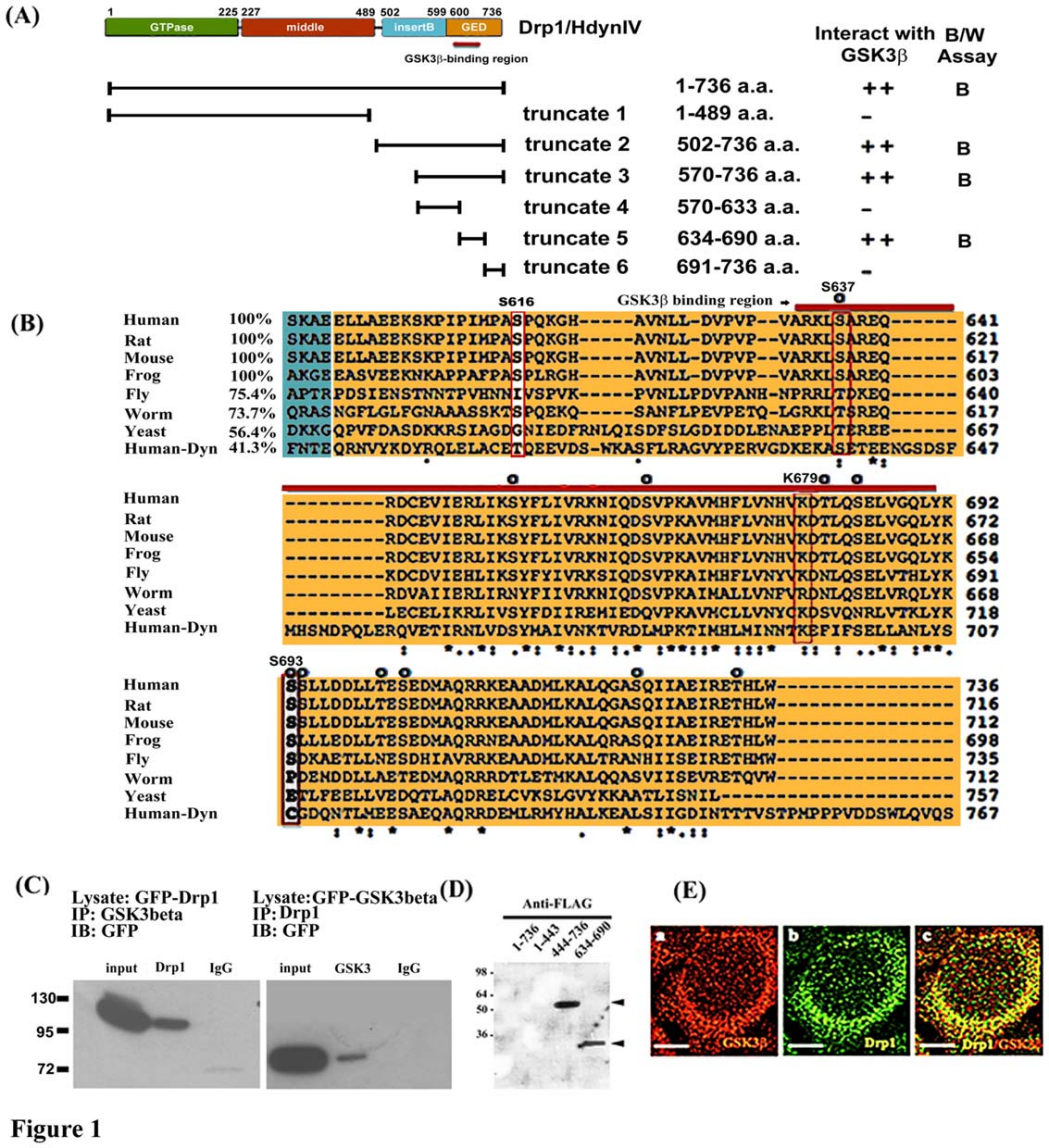
Interaction and co-localization of Drp1/Hydn IV with GSK3beta. (A) Schematic diagram showing the domain structure of Drp1 as previously described [7] and relative positions of truncated Drp1 constructs used in this study. Yeast two-hybrid assays showing interactions of various Drp1 constructs as bait (pAS2-1) with GSK3beta as prey (pACT-2) as indicated. Blue (B) and white (W) assay was applied for reporter gene beta-galactosidase expression. Strength of interaction was assayed by beta-galactosidase and HIS3 induction as described previously by Hong et al. [5]. (B) Specific binding region of Drp1 with GSK3beta is highly conserved. Multiple alignment of Drp 1 GED domain (colored in orange) was performed using ClustalW2 by which seven Drp1 homologues and human Dynamin 1 were aligned. Note the very high conservation in the GSK3 binding domain (BD, indicated by red bar) in Drp1 but not Dynamin 1 and Ser or Lys residues at positions corresponding to human Drp1 S637, K679, and S693 as indicated by red or white rectangles. Compared to human Drp1, the percentage of amino acid identity corresponding to each BD in GED is listed in front of the sequence. The prospective phosphorylated Ser/Thr residues are indicated by “O”. (C) Total cell lysates were prepared from pEGFP C1-Drp1, pEGPC1-GSK3beta overexpressed 293 cells after transient expression for 24 hours, and subjected to co-immunoprecipitation (IP) assays with anti-GSK3 (left panel), anti-Drp1 (right panel), respectively. The anti-GFP antibody was used to detect the exogenous expressed GFP-tagged proteins in Western blotting (as indicated) which has been described in Materials and Methods. Data are representative of three independent experiments. The IP-Drp1 and IP-GSK3 represents the co-precipitated protein respects to antibodies used (D) The binding fashion of Drp1_634–690_ and Drp1_444–736_ fragment with GSK3beta was confirmed by far-Western blotting. Recombinant His-tagged fusion protein, either Drp1 full-length or truncated fragments of Drp1, was separated on 12% SDS polyacrylamide gel. Proteins were transferred into PVDF and incubated with HeLa cell lysate containing FLAG-tagged GSK3beta. Arrowhead indicates binding. (E) Co-localization study was performed by immunocytochemistry staining with confocal fluorescent microscopy as described in Materials and Methods. At 24 hours, 293 cells were stained with anti-GSK3beta antibody to detect endogenous GSK3beta (red, frame a). Frame b is the localization of endogenous Drp1 (green). Frame c is the merged image of the Drp1 and anti- GSK3beta stained cells. Bar = 5 µm.

Drp1 is one of the dynamin related proteins, which is a large protein reported to be comprised of an amino-terminal GTPase domain, middle domain, insert B and a GTPase effector domain (GED) as shown in Figure 1A [7]. Drp1 is involved in several important mitochondrial events, including shape, size, distribution, remodeling, and maintenance of mitochondria in mammalian cells [8]. Mitochondrial morphology is one of the important issues in determining cell fate and needs to be precisely controlled in a living cell. Previous studies also revealed that Drp1 is crucial for opposing the fission/fusion machinery of mitochondria and affects the balance of mitochondrial dynamics [9], [10]. It was also found that increasing Dnm1 (yeast Drp1 homologue) self-assembly/oligomerization mirrors its elevated GTPase hydrolytic activity, resulting in mitochondrial fragmentation [11].

Regarding the functional regulation of Drp1, post-translational modifications such as sumoylation, S-nitroylation, ubiquitination, and phosphorylation are thought to be an important part of its regulation [12], [13]. Preferentially, Drp1 Ser637 was first identified as a phosphorylation site by protein kinase A (PKA) and as a dephosphorylation site by calcineurin [14]–[16]. Drp1phosphorylation at Ser637 site leads to elongated mitochondrial morphology; in contrast, Han et al. (2008) reported that phosphorylation of the Ser637 site by CaMK1α promoted mitochondrial fission with binding to Fis1 [17]. However, a recent report suggests that Mff is an essential factor for mitochondrial recruitment of Drp1 [18]. Unlike Drp1 phosphorylation at Ser637 by PKA, phosphorylation at Ser616 of Drp1 (equivalent to Ser585 of rat Drp1) by CDK1/Cyclin B induces mitochondrial fission during mitosis [14], [19]. Additionally, phosphorylation of Drp1has been implicated in controlling mitochondrial fission and mitophagy induction [20]. Although phosphorylation of two sites (Ser616 & Ser637) regulated by kinases influences mitochondrial recruitment of Drp1, pointing to opposing functional and morphological effects [12]–[17], [19], the physiological role of such phosphorylation events still remains paradoxical. It should be also noted that phosphorylation at Ser637 by PKA or by a phosphomimetic mutant induces elongated mitochondria and protects cells against pro-apoptotic stimuli [14], [15]. Given our previous report that GSK3beta interacts with and phosphorylates Drp1 [5], [6], we attempted to identify the functional consequences of GSK3beta-mediated phosphorylation of Drp1 at the Ser693 site in addition to Ser616 and Ser637 located within the GED domain. In this study, we clearly demonstrated that GSK3beta-mediated phosphorylation at the Ser693 site induced an elongated mitochondrial morphology and exhibited an anti-apoptotic effect against H_2_O_2_-induced apoptosis rather than inducing autophagy.

## Results

### Interaction and co-localization of Drp1 with GSK3beta

To determine the binding region of GSK3beta on Drp1, we performed yeast two-hybridization, co-immunoprecipitation, gel overlay assay and co-localization experiments. In yeast two-hybridization, we created different truncated Drp1/Hydn IV to map its binding region with GSK3beta. The results showed that only the wide-type and truncate 2, 3, and 5 fragments interact with GSK3beta, and not the truncate 1, 4 and 6 (Figure 1A). This data suggested the region Drp1_634–690_ as a possible GSK3beta binding domain. Protein sequences of Drp1 orthologies among different species were aligned by Clustal W2 (Figure 1B). Human, rat, mouse, and frog showed 100% identity and fly showed 75% identity to human Drp1 on the GED domain. Highly conserved features were visible for all Drp1 homologues, but not dynamin1, which only retains 41.3% amino acid similarity when compared with human Drp1. This finding may indicate that the property of GSK3beta-interaction is only conserved down to yeast homologue. By observing amino acid residues within the GED domain of Drp1 across different species, 5 conserved Serine/Threonine sites were located in the GSK3beta-binding region (Ser637, Ser653, Ser665, Thr681, and Ser684), and 6 conserved Serine/Threonine (Ser/Thr) sites were located outward from the GSK3beta-binding region (Ser693, Ser694, Thr701, Ser724, and Ser733). Using co-immunoprecipitation, the overexpressed GFP-Drp1 and GFP-GSK3beta in 293 cells can be successfully co-immunoprecipitated with antibody against GSK3beta and Drp1, respectively. We clearly detected GSK3beta interaction with Drp1 protein in the blot (Figure 1C). The gel overlay assay also suggested that two truncated Drp1 fragments, Drp1_444–736_ and Drp1_634–690_, were able to interact with FLAG-GSK3beta, which can be detected by anti-FLAG antibody directly against GSK3beta (Figure 1D). However, the failure of full length Drp1 to interact with GSK3beta may be due to the problem of protein folding attributed to using a prokaryotic expression system. Furthermore, using confocal fluorescent microscopy, both endogenous Drp1 and GSK3beta proteins were co-localized predominantly in the cytosol (Figure 1E). Taken together, our data suggested that Drp1 directly interacts with GSK3beta *in vivo* and *in vitro* and Drp1_634–690_ is the required region for GSK3beta binding.

### Drp1 Ser693 is a GSK3beta phosphorylation site

**Figure 2:**
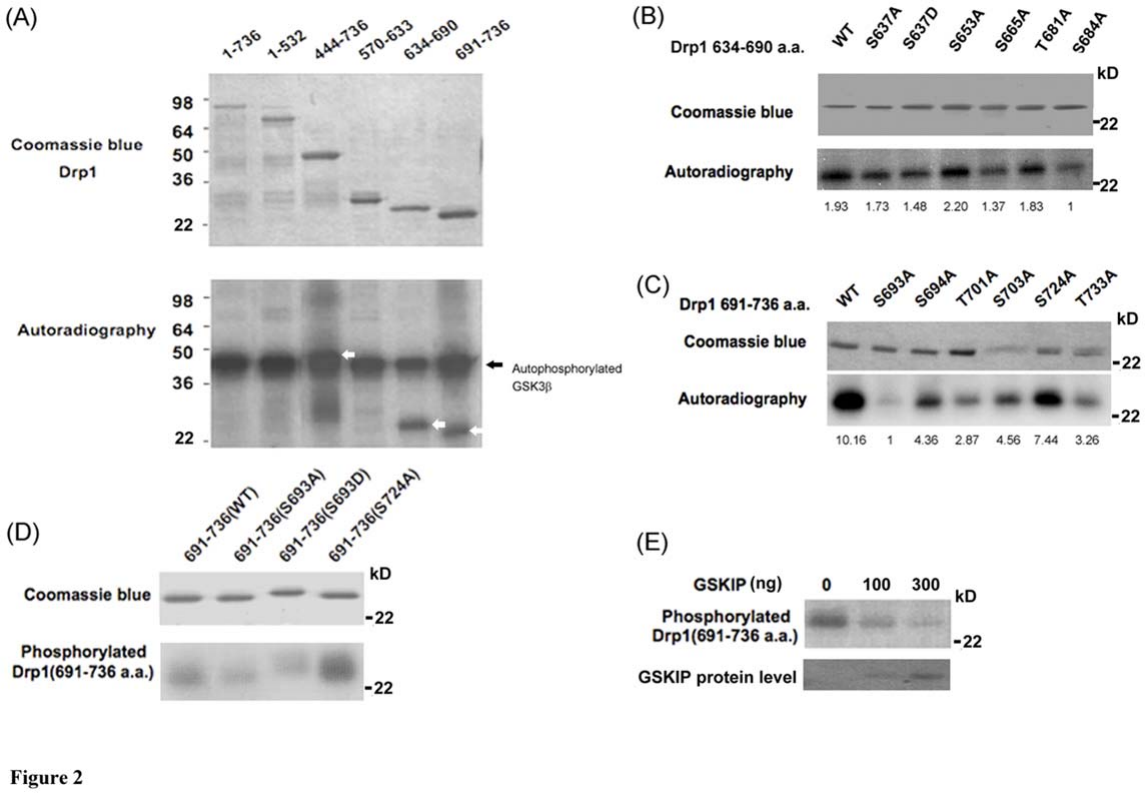
Drp1 is phosphorylated by GSK3beta at Ser693. Drp1 is phosphorylated by GSK3beta. (A) Purified GSK3beta was used in an *in vitro* kinase assay to detect whether and which truncated His-tagged Drp1 proteins can act as a substrate to be phosphorylated by GSK3beta. The upper panel is a coomassie blue stained blot showing the His-tagged Drp1 proteins were successfully expressed. The lower panel indicates GSK3beta autophosphorylation (arrow) and phosphorylation of truncated His-Drp1 (empty arrow). (B) *In vitro* kinase assay to determine the GSK3beta phosphorylation site using site-directed mutagenesis technique. Numbers indicate the mutated residue within the Drp1_634–690_ fragment. The upper panel is a coomassie blue stained blot showing that His-tagged Drp1_634–690_ wild-type and mutants were expressed. The lower panel indicates the phosphorylation level with respect to His-tagged Drp1_634–690_ wild-type and different mutants. (C) Numbers indicate the mutated residue within the Drp1_691–736_ fragment. The upper panel is a coomassie blue stained blot showing that His-tagged Drp1_691–736_ wild-type and mutants were expressed. The lower panel indicates the phosphorylation level with respect to His-tagged Drp1_691–736_ wild-type and different mutants. (D) Both S693A and S693D Drp1 mutants were confirmed to lose their GSK3beta-mediated phosphorylation *in vitro*. The upper panel is the coomassie blue staining of His-tagged mutated Drp1 fragments. Numbers indicate the mutated residue within the Drp1_691–736_ fragment. The lower panel indicates the phosphorylation of mutated His-tagged Drp1. (E) The specificity of GSK3beta-mediated phosphorylation was tested on Drp1_691–736_ fragments. GSK3beta inhibitor, GSKIP, was added in kinase assay with different doses and the effect on the phosphorylation of Drp1_691–736_ is shown. The coomassie blue image of GSKIP protein as loading/input control was shown in the lower panel.

Our previous results suggested that Drp1 interacts with GSK3beta and acts as a substrate of GSK3beta [6]. However, the exact phosphorylation site and the functional consequence of GSK3beta-mediated phosphorylation of Drp1 are yet to be identified. Therefore, we performed an *in vitro* kinase assay of GSK3beta which incubated the recombinant Drp1 variants that were expressed by the pET protein expression system with GSK3beta. Subsequently, we determined the variation between these Drp1 variants in terms of their role as a substrate to be phosphorylated by GSK3beta. In this experiment, we found that the fragments with respect to Drp1_634–690_ and Drp1_691–736_ are two relatively small peptides serving as phosphorylating substrates of GSK3beta and that Drp1_444–736_ can also be phosphorylated by GSK3beta (Figure 2A). Likewise, the full-length Drp1 was not found to be phosphorylated by GSK3beta, which indirectly supported our suspicion that the problem of protein folding would be an obstacle for Drp1 interaction with GSK3beta. GSK3beta is a Ser/Thr protein kinase and there are eleven conserved Ser/Thr sites on the Drp1 GED domain, including Ser637, Ser653, Ser665, Thr681, Ser684, Ser693, Ser694, Thr701, Ser703, Ser724 and Thr733, which may be phosphorylated by GSK3beta (Figure 1B). To further define the possible phosphorylation sites within Drp1_634–690_ and Drp1_691–736_, the site-directed mutagenesis technique was used to convert these possible phosphorylation sites into alanine. According to the experimental evidence from the i*n vitro* kinase assay, the S665A and S684A Drp1_634–690_ mutants were found to moderately decrease their phosphorylation by GSK3beta compared to the other Drp1_634–690_ mutants (Figure 2B). In comparison with wild-type Drp1_634–690_, a 1.41- and 1.93-fold decrease in GSK3beta-mediated phosphorylation level was found for S665A and S684A, respectively. Of the Drp1_691–736_ mutants, S693A, T701A and T733A were all found to have an evident decrease in their GSK3beta-mediated phosphorylation level. Compared with the wild-type Drp1_691–736_ group, a 10.16-, 3.54-,3.12-fold decrease was found for S693A, T701A and T733A mutant, respectively. The S693A Drp1_691–736_ mutant was the most effective in down-regulating the GSK3beta-mediated phosphorylation level compared to the other Drp1_634–690_ and Drp1_691–736_ mutants (Figure 2B & 2C). It is of interest that the human Drp1 Ser693 site is highly conserved among different species (Figure 1B). Considering the high conservation of the Ser693 site across different species as well as that Ser693 highly affects the GSK3beta-mediated phosphorylation of Drp1, we speculated that Ser693 may be involved in the biological function of Drp1 and that this GSK3beta-mediated phosphorylation event is also conserved. To further explore the roles of the GSK3beta-mediated phosphorylation of Drp1, the Drp1_690–736_ fragment was subjected to site-directed mutagenesis to obtain a mimetic phosphorylated mutant (S693D) of Drp1 in addition to a dephosphorylated (S693A) mutant. Both of these two Drp1 mutants were confirmed to lose their GSK3beta-mediated phosphorylation *in vitro* (Figure 2D) compared to S724A and wild-type Drp1_690–736_. To verify the specificity of the phosphorylation event, we applied the GSK3beta inhibitor [21], GSK3beta interacting protein (GSKIP), to abolish the GSK3beta-mediated phosphorylation. As the result shows in Figure 2E, GSK3beta-mediated phosphorylation was gradually inhibited by GSKIP in a dose-dependent manner. These results clearly indicate that GSK3beta binds to the fragment corresponding to Drp1_634–690_ and phosphorylates Drp1 at the Ser693 site.

### Drp1 S693 and K679 locate in GED and do not affect inter-/intra- molecular interactions, but K679 determines GSK3beta binding

**Figure 3:**
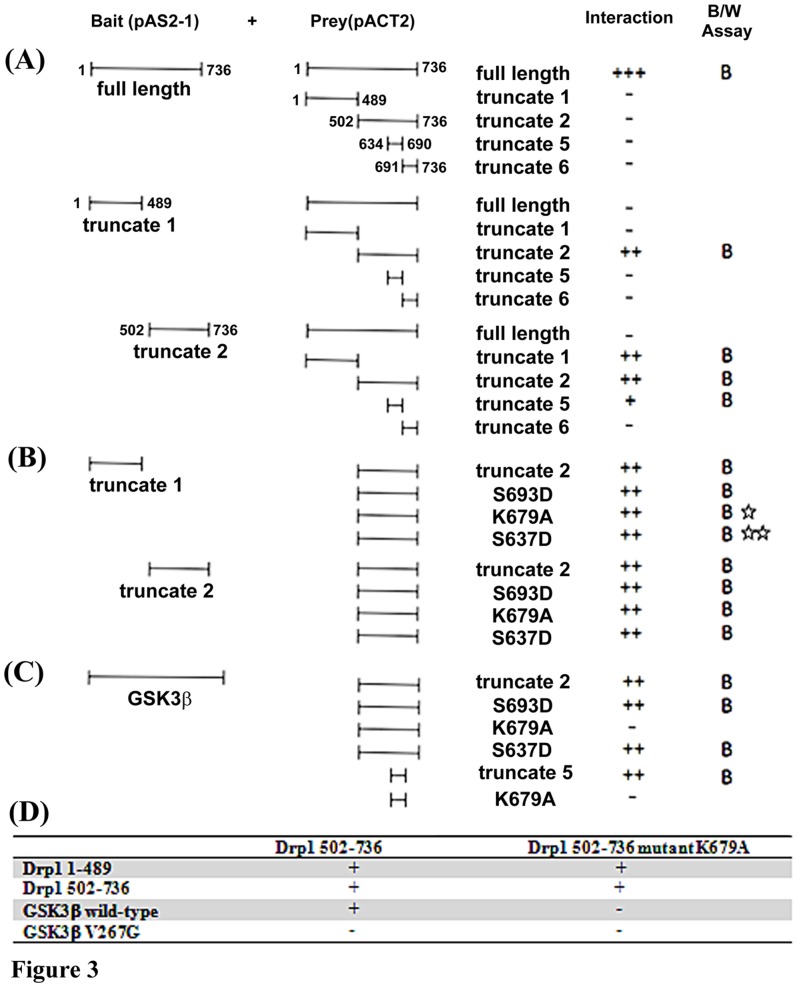
Yeast two-hybrid assay identifying Drp1 inter-/intra- interaction and residue responsible for GSK3beta-Drp1 binding. The design of the yeast two-hybrid assays showing interactions of various bait (pAS2-1) or prey (pACT2) constructs is indicated. The strength of interaction was assayed as described in Methods. beta-galactosidase and HIS3 induction was quantified as described in Materials and Methods. The result of the interaction was shown by “+” or “−”. (A) The inter-molecular interaction of Drp1 was tested by using full-length Drp1 as bait and various truncated Drp1 as prey to verify their interacting ability. The intra-molecular interaction of Drp1 was tested by using truncate 1 or 2 of Drp1 as bait and various truncated Drp1 as prey to verify their interacting ability. (B) Drp1 truncated fragments with point-mutation were tested to verify their interaction with Drp1 truncate 1 or 2. Some of our results were inconsistent with previous reports [14], [23] and are indicated as “☆”and “☆☆”, respectively (C). Some Drp1 truncate 2 mutants were tested for their possible interaction with GSK3beta. (D) Matrix of yeast two-hybrid assays showing interactions of various Drp1 N- and C-terminal deletion fragments, GSK3beta wt and GSK3beta V276G mutant in bait constructs, tested against C-terminal Drp1 prey constructs with or without K679A mutation as indicated. The result of interaction was shown as “+” or “−”.

The GTPase Effect Domain (GED) has been suggested to regulate the GTPase activity of dynamin related proteins via inter-/intra-molecular interactions [22]. We wondered whether GSK3beta-mediated phosphorylation at Ser693 resulted in any consequences on Drp1 inter-/intra-molecular interaction similar to those reported with respect to phosphorylation events at S637 and K679 [14], [23]. The yeast two-hybrid system was utilized to test the functional consequences corresponding with GSK3beta-mediated phosphorylation. First, we tried to identify the minimal required regions responsible for the inter-/intra-molecular interaction between Drp1 monomers. The interaction data displayed in Figure 3A suggested that no matter whether the Drp1 truncate 1 fragment (Drp1_1–489_) was used as prey or bait protein in the yeast two hybrid system, it can interact with Drp1 truncate 2 fragment (Drp1_502–736_). This finding suggests that Drp1_1–489_ is important for intra-molecular interaction. In addition, the entire GED domain is also required for the inter-molecular interactions (truncate 2 interact with truncate 1, 2 and 5, but not 6). Altogether, these data suggest that Drp1_634–690_ (truncate 5) might be important to both the inter-/intra-interactions of Drp1 assembly (Figure 3A). Surprisingly, when we tried to answer whether these interactions would be abolished by point mutations (S693D, S637D or K679A) in the truncate 2 fragment (Drp1_502–736_), the results showed no differences between these three Drp1 mutants and wild-type Drp1. Our data indicated that S693D, S637D and K679A are unlikely to impair intra-molecular interaction and Ser693 may not be involved in Drp1 inter-/intra-molecular interactions (Figure 3B). Nevertheless, it should be noted that our data are in conflict with other studies in which S637 or K679 mutants were reported to interfere with the intra-molecular interaction of Drp1 monomers [14], [23] (Figure 3B, asterisks). To confirm the negative finding that GSK3beta-mediated phosphorylation at Ser693 is not involved in inter-/intra-molecular interactions of Drp1, we re-performed an interactive assay to determine whether GSK3beta may interact with Drp1 fragments harboring point-mutations in the GED domain. Intriguingly, the results showed that GSK3beta is only unable to interact with K679A mutant, but not the wild-type Drp1 (truncate 2 and 5) and S693D mutant (Figure 3C). In the results from a previous study, the interaction between Drp1 and GSK3beta was affected by GSK3betaV267G, but not GSK3betaY288F mutant [24]. Using a yeast two-hybrid system, our data further confirm that the V267 of GSK3beta together with K679 of Drp1 is a critical residue for GSK3beta-Drp1 interaction (Figure 3 C & D). In conclusion, these data indicate that Drp1 might function as a novel substrate for GSK3beta.

### Drp1 Ser693 is involved in regulating its GTPase activity

**Figure 4:**
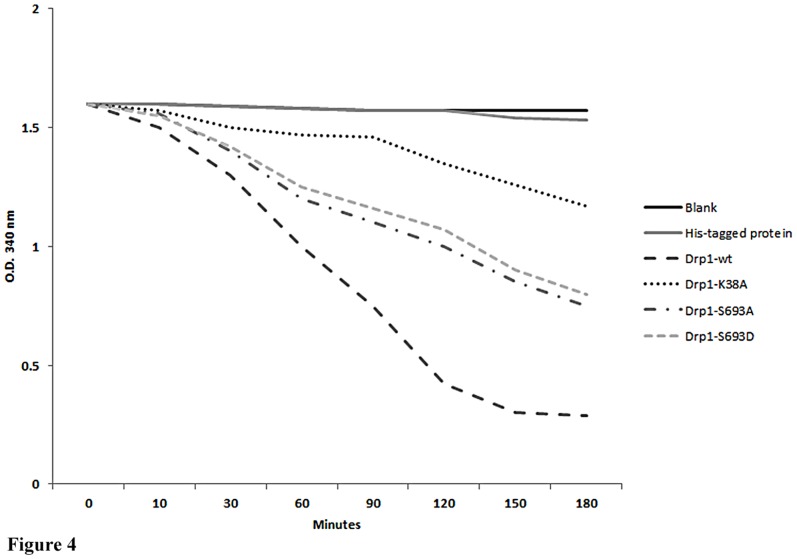
GTPase hydrolysis activity of Drp1mutants. A GTPase hydrolysis activity assay was performed followed the procedures published by Ingerman and Nunnari [41]. *E. coli* were transformed by Drp1 mutant plasmids and 15 µg of cell lysate was applied to the assay. O.D. 340 was measured to reflect the constant of the NADH. The slope reflects the consumption of NADH. The data are representative of three independent experiments and are shown as mean values ± SD.

Our data suggested that Ser693 is not involved in the inter-/intra-molecular interactions of Drp1. However, the GED in dynamin has been proposed to regulate dynamin, which functions as a GAP (GTPase activating protein) to stimulate GTP hydrolysis after dynamin assembly [22]. Therefore, we investigated whether phosphorylation of S693 directly affects the GTPase activity. We used a phosphomimetic substitution, S693D, to compare its GTPase activity with that of S693A and the GTPase-dead mutant (K38A). Through *in vitro* GTPase assays, we found that phosphomimetic His-Drp1 S693D exhibited the same decreased GTPase activity as the S693A and K38A mutants (Figure 4). The data suggested that the phosphomimetic S693D on Drp1 decreases GTPase activity even though Ser693 was not found to impair the inter-/intra-molecular interaction of Drp1 in this study.

### Phosphorylation of Drp1 at Ser693 inhibits mitochondrial fission

**Figure 5:**
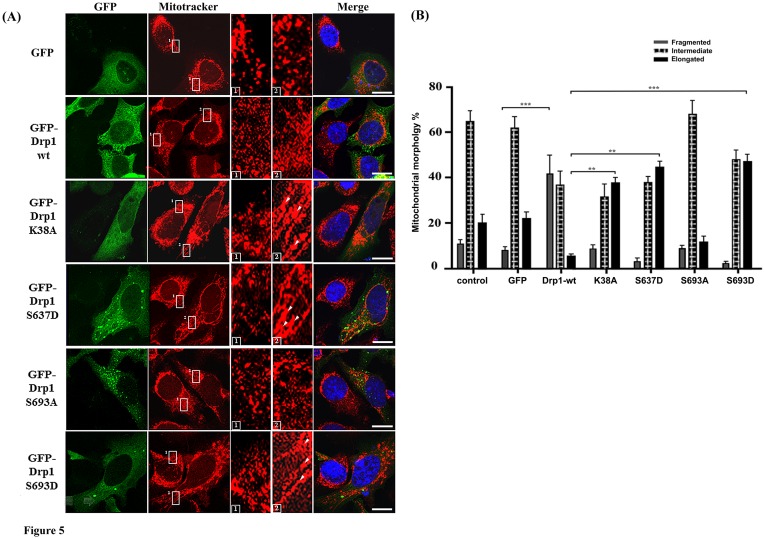
Mitochondrial dynamics of HeLa cells with Drp1 wt and mutants. (A) HeLa cells were transfected with GFP-tagged Drp1 wt or other mutants for 24 hours. Mitochondrial morphology was observed by confocal fluorescent microscope with Mitotracker dye. Cell nuclei were counter-stained by DAPI. Insets are magnifications of the Mitotracker signal at the indicated areas. Inset 1 represents the non-transfected cells, and inset 2 indicates the transfected cells. Indications (white arrows) represent typical elongated mitochondria morphology. (B) Statistical result of mitochondrial morphology. After 24 hours, over 100 transfected cells were categorized into 3 groups depending on mitochondrial morphology. *p<0.05, **p<0.01, ***p<0.001.

**Figure 6:**
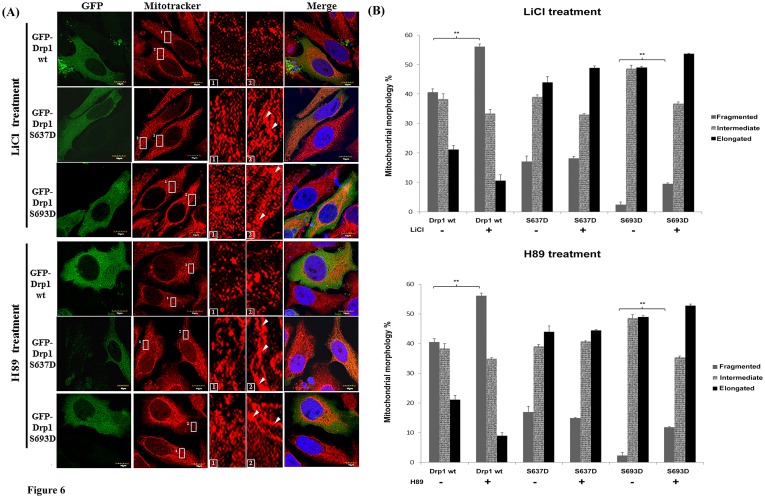
Mitochondrial dynamics of HeLa cells with Drp1 wt and mutants and were treated with LiCl and H89. (A) HeLa cells were transfected with GFP-tagged Drp1wt or other mutants for 24 hours. Then cells were treated with 10 mM LiCl and 10 µM H89 for another 24 hours. Mitochondrial morphology of HeLa cells was observed by staining with Mitotracker under confocal microscopy. Cell nuclei were counter-stained by using DAPI. Insets are magnifications of the Mitotracker signal at the indicated areas. Inset 1 represents the non-transfected cells, and inset 2 indicates the transfected cells. Indications (white arrows) represent typical elongated mitochondria morphology. (B) Statistical result of mitochondrial morphology. After 24 hours treated with inhibitors (upper: LiCl, lower: H89), over 100 transfected cells were categorized into 3 groups depending on mitochondrial morphology. *p<0.05, **p<0.01, ***p<0.001.

To elucidate whether the Drp1phosphorylation by GSK3beta affects mitochondrial morphology, we transfected HeLa cells with constructs expressing different mimetic phosphorylated Drp1 mutants. Five different mutations (K38A, S637D, K679A, S693A and S693D) were tested to evaluate the biological functionalities involved in regulating mitochondrial morphology, especially with respect to reported disruptions of Drp1 functions in controlling inter-/intra-molecular interaction (S637D). The GFP-expressing cells were examined for their protein expression (see Figure S1) and were further stained by Mitotracker to observe their mitochondrial morphology (Figure 5A). Overexpression of GFP-Drp1 wild-type significantly increased the mitochondrial fragmentation (40% vs. 8.9% as compared to the GFP group, p<0.001). Overexpression of the phosphomimetic S693D mutant showed an increase of elongated mitochondria (48.7% vs. 23.5% as compared to the GFP group). A similar morphological shift of mitochondria was also found in the K38A (41.3% vs. 23.5% as compared to the GFP group) and S637D (46.5% vs. 23.5% as compared to the GFP group) groups, but not the S693A mutant (17.8% vs. 23.5% as compared to the GFP group) (Figure 5B). By comparing the portion of elongated mitochondria between the Drp1 mutant and Drp1 wild-type group, the S693D group showed the most significant difference (p<0.001) followed by the S637D, K38A (p<0.01), and K679A group (Figure S2). The S693A group does not cause significant variation on mitochondrial morphology as shown in Figure 5B. To further confirm the GSK3beta-mediated phosphorylation of Drp1 at the Ser693 site as well as PKA-mediated phosphorylation at the Ser637site, LiCl and H89 were used as inhibitors to block GSK3betaand PKA signaling respectively in HeLa cells. The elongated mitochondrial morphology was significantly reversed to a fragmented one in the S693D group (p<0.001), but not in the S637D group. Expectedly, since S693D is a phosphomimetic mutant, only a portion of the elongated mitochondria switched to a fragmented phenotype after either LiCl or H89 treatment in the S693D group when comparing the variation of fragmented mitochondria between the untreated Drp1wt group and inhibitors-Drp1 wt group (Figure 6A & B). Taken together, we found that GSK3beta-mediated phosphorylation of Drp1 at the Ser693 site regulates mitochondrial morphology through altering the GTPase activity of Drp1 even if the Drp1 self-assembly is not changed.

### Overexpressed Drp1 S693D leads to resistance to hydrogen peroxide -induced mitochondrial fragmentation and ensuing apoptosis, but did not induce autophagy

**Figure 7:**
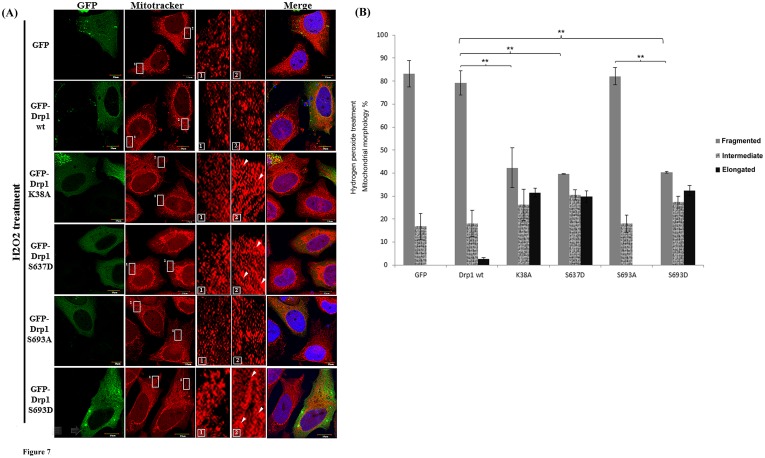
Mitochondrial dynamics of HeLa cells with Drp1 wt and mutants treated with H_2_O_2_. (A) HeLa cells were transfected with GFP-tagged Drp1wt or other mutants for 24 hours. Then cells were treated with 500 µM H_2_O_2_ for another 24 hours. Mitochondrial morphology of HeLa cells was observed by staining with Mitotracker under confocal microscopy. Cell nuclei were counter-stained by using DAPI. Insets are magnifications of the Mitotracker signal at the indicated areas. Inset 1 represents the non-transfected cells, and inset 2 indicates the transfected cells. Indications (white arrows) represent typical elongated mitochondria morphology. (B) Statistical results demonstrated mitochondrial morphology of HeLa cells with or without Drp1 expression under H_2_O_2_ treatment for 24 hours; over 100 transfected cells were categorized into 3 groups depending on mitochondrial morphology. **p<0.001.

To investigate the role of Drp1-associated elongated mitochondrial morphology, we therefore further examined cell lines expressing Drp1 and other Drp1 mutants for their susceptibility to the apoptotic stressor H_2_O_2_ since the elongated mitochondrial morphology has been linked to a protective effect against both apoptosis and autophagy. After treating HeLa cells with 500 µM H_2_O_2_ for 24 hours, the mitochondrial networks with respect to GFP alone, GFP-Drp1 wild-type (wt) and the S693A group did show a phenotype of fragmentation, in which almost 80% of the mitochondria of cells underwent fragmentation (Figure 7A & 7B). In contrast, overexpression of GFP-Drp1 K38A, S637D and S693D prevented cells from undergoing H_2_O_2_-induced mitochondrial fragmentation (Figure 7A & 7B). By comparison with the fragmented mitochondria of the Dr1p wt group, the K38A, S637D and S693D group all showed significant lower numbers of fragmented mitochondria (p<0.01). Likewise, the S693D group also exhibited a significantly greater level of fragmented mitochondria than the S693A group (p<0.01) as shown in Figure 7B.

**Figure 8:**
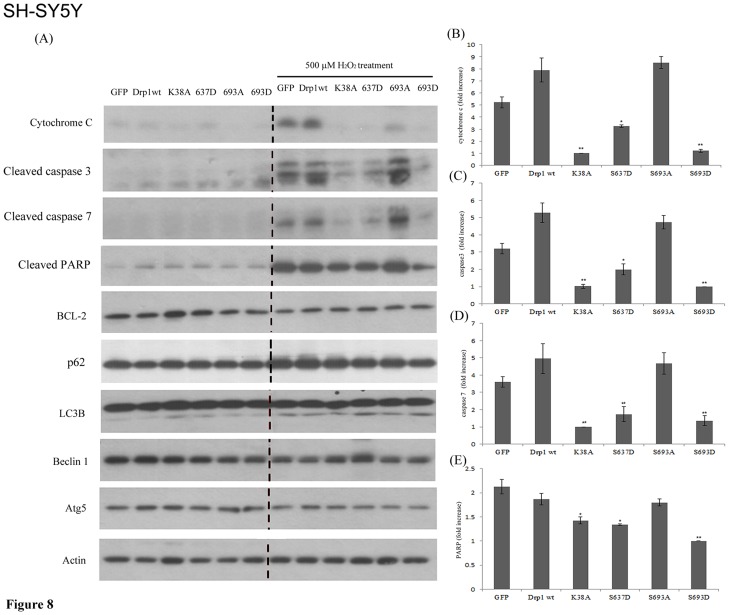
Overexpressed Drp1 S693D can protect against H_2_O_2_-induced mitochondrial fragmentation and ensuing apoptosis but does not induce autophagy. SH-SY5Y cells were transfected with GFP alone, GFP-tagged Drp-1 wild-type and other mutants for 24 hours. Then cells were treated with 500 µM H_2_O_2_ for another 24 hours, and were lysed and detected by Western blotting using anti-cytochrome c, cleaved-caspase 3, cleaved-caspase 7, cleaved-PARP, -Bcl2, -LC3, p62, -Beclin 1 and -Atg5 antibody, respectively. beta-actin served as a protein loading control. The data are representative of three independent experiments.

Since ectopic expressed GFP-Drp1 mutants (K38A, S637D, and S693D) exert a protective effect for HeLa cells to combat H_2_O_2_-induced mitochondrial fragmentation, we further dissected whether and which apoptotic, anti-apoptotic and autophagy-related protein expressions are affected consequent to the elongated mitochondria phenotype induced by ectopic expressed Drp1 or Drp1 mutants in HEK293 and SH-SY5Y cells with or without H_2_O_2_ treatment. First, the Drp1 wt and mutants were transfected into HEK293 and SH-SY5Y cells, respectively. Afterwards, immunoblotting was used to detect the protein expression level of apoptotic and anti-apoptotic genes. In SH-SY5Y cells, after insult with 500 µM H_2_O_2_ for 24 hours, the K38A, S637D and S693D group showed a significantly lower level of cytochrome c, caspase-3, -7 and PARP induction compared to the Drp1 wt group (Figure 8A–E). In contrast, no inter-group difference was found for Bcl-2 (an anti-apoptotic protein) or LC3B, p62, Atg5 and Beclin-1 (autophagy-related proteins). A similar pattern of protein expression consequent to ectopic expression of Drp1 mutant and H_2_O_2_ insult was found in HEK293 cells (Figure S3). GSK3beta-mediated phosphorylation at the Ser693 site and PKA signaling are all associated with the elongated mitochondria; in addition, the inhibition of PKA signaling is insufficient to reverse the elongated mitochondrial morphology caused by ectopic expression of Ser637D Drp1 mutant. Taken together, these results indicated that GSK3beta-mediated phosphorylation at Drp1 Ser693, like the Drp1 Ser637 that is phosphorylated by PKA, leads to elongated mitochondrial morphology and protects HEK293 and SH-SY5Y cells against H_2_O_2_-induced mitochondrial fragmentation and ensuing apoptosis by down-regulating cytochrome c, caspase-3, -7 and PARP activities. Ectopic expressed Drp1 S693D, K38A and S637D mutant increased the ratio of elongated mitochondria and may also be associated with preventing H_2_O_2_-induced apoptosis.

## Discussion

In the present study, we reported the possible functional roles of GSK3beta interaction as well as GSK3beta-mediated phosphorylation of Drp1 at the GED domain. We successfully identified the GSK3beta phosphorylation site at Ser693 of Drp1. We demonstrate that GSK3beta binds to Drp1 via 634–690 residue and phosphorylates Ser693 in the GED domain. Using a mimetic phosphorylated mutant of S693D, we also found that expressed S693D Drp1 mutant resulted in decreased GTPase activity *in vitro*, decreased mitochondrial fission, and caused elongated mitochondrial morphology. Finally, we concluded that GSK3beta-mediated phosphorylation at Ser693 may be associated with elongated mitochondrial morphology and correlated to acquired resistance against H_2_O_2_-induced apoptosis via down-regulating cytochrome c, caspase-3, -7 and PARP activities, but does not induce autophagy

In this report, a new Drp1 phosphorylation site, Ser693, was identified and phosphorylation at this site was shown to lead to elongated mitochondrial morphology. Although the role of GED in dynamin assembly is widely accepted to be correlated to its GTPase activity, the mechanism by which mutation in GED leads to conformational change of Drp1 and cooperatively increases GTPase activity remains uncertain. Chang et al. found that the S637D mutant of Drp1 impairs intra-molecular interactions of the GED domain and middle domains, indicating that S637D mutation may be associated with a conformational change in the GED that interferes with the inter-domain interactions of Drp1 monomer [14]. Contradictorily, in our study we were unable to replicate the findings of Chang et al. that S637D mutation does not affect the interactions between Drp1_1–489_ and Drp1_502–736_ or between two Drp1_502–736_ fragments in a yeast two hybrid system. Moreover, the K679A and S693D Drp1 mutations also do not interfere with inter-/intra-molecular interactions of Drp1 truncated fragments (Figure 3). The inconsistency between our results and previous reports may be due to variations in the assay system used [14], [23]. Moreover, Chang et al. performed a semi-quantified study on a yeast-two-hybrid system and simply indicated the so-called decreased intra-interaction from “+++” to “+” [14], [23]. In our system, we did not quantify the level of interaction in the same way and thus our results are not comparable with theirs. However, to confirm and verify our results using a yeast two-hybrid system, we further tested the interaction between Drp1_502–736_ K679A mutant, Drp1_1–489_, Drp1_502–736_ and GSK3beta (Figure 3D). Interestingly, GSK3beta-Drp1 interaction was abolished in the K679A group, which is located in our defined GSK3beta-binding region (Figure 1A and 3C). The Drp1_634–690_ is responsible for GSK3beta interactions, and the K679 of Drp1 and V267 of GSK3betaare critical residues for their interaction. Moreover, the Ser693 is not responsible for the GSK3beta interaction. Our data, in contrast to previous reports, suggests that the K679 site does not affect the interactions of the GED-GED (inter-molecule) corresponding to Drp1_502–736_ and/or the GED-GTPase domain (intra-molecule) refers to Drp1_1–489_ and Drp1_502–736_. These two kinds of interactions are rationalized as two major molecular mechanisms consequent to the decrease in GTPase activity of Drp1. Therefore, our finding that Ser693 is only responsible for GSK3beta-mediated phosphorylation, but not involved in the inter-/intra-molecular interactions of Drp1 monomers, may be indirectly supported by these data. Obviously, the Drp1 K679 is more likely to be important for GSK3beta binding and Ser693 is a GSK3beta phosphorylation site.

**Figure 9:**
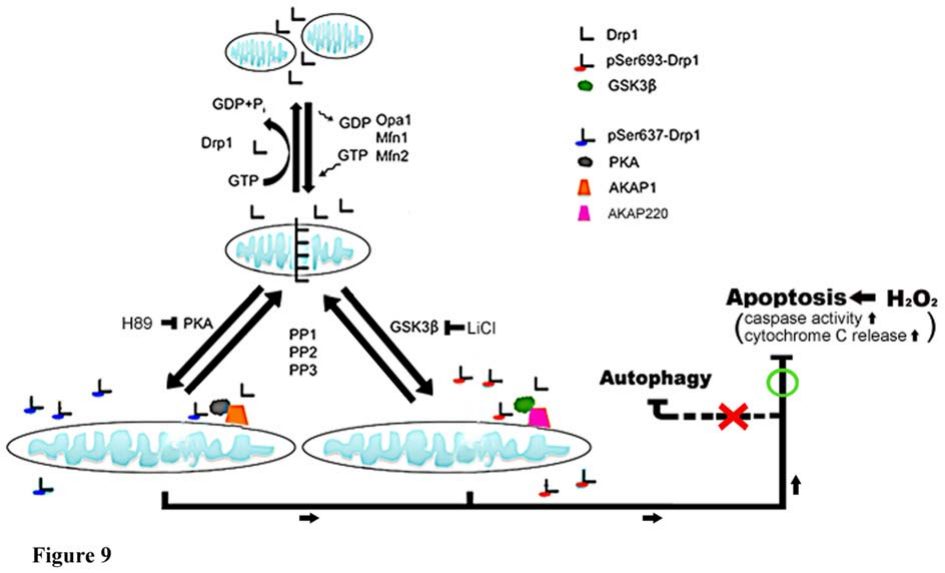
Model represents both GSK3β- and PKA-mediated Drp1 phosphorylation induction of mitochondrial elongation which subsequently causes acquired resistance to H_2_O_2_-induced apoptosis rather than inducing autophagy. Mitochondria dynamics regulate the GTPase hydrolysis activity of proteins (Drp1, Opa1, Mfn1 and 2) resulting in mitochondrial fission or fusion. In this model, two Drp1 phosphorylation sites could serve a regulatory function, including phosphorylation by PKA/AKAP1 on Ser637 [39] or by GSK3β on Ser693 (as shown in this study), leading to diminished mitochondrial fission resulting in mitochondrial elongation. GSK3β might be recruited to mitochondria through AKAP220 and be dephosphorylated by PP1, 2, and 3 [40]. Such mitochondrial morphological changes could also result in cell fate determination. Mitochondrial fission is involved in the initiation of apoptosis, whereas mitochondrial fusion may induce autophagy. Both phosphorylation events occurring at S637 and S693 cause elongated mitochondrial morphology and lead to acquired resistance to H_2_O_2_-induced mitochondrial fragmentation and ensuing apoptosis via down-regulating cytochrome c release, capase-3, -7 and PARP activations rather than inducing autophagy.

Drp1 exhibits a basal level of GTP hydrolysis that is enhanced by self- assembly/oligomerization. GED in Drp1 is required for activating its GTPase activity, though the underlying mechanisms are still poorly understood. The function of GED in regulating dynamin has been suggested via two distinct ways. First, it may regulate GTPase activity through self-assembly or highly ordered GED-GED interaction. Recently, evidence from protein structural studies has revealed that GED may biophysically serve as a docking site for GTPase binding and exerts stimulatory effects [25]. Second, the GED directly acts as a GAP (GTPase activating protein) to stimulate GTP hydrolysis after dynamin assembles [22]. In our study, no impairment of the interaction of GED-GED and mutated GED (S693D) with the GTPase domain was found, suggesting that inter-/intra-molecular interaction of this Drp1 phosphomimetic mutant is somehow protected (Figure 3C). However, according to our findings showing Figure 4, both S693A and S693D are with a deceased GTPase activity. We suggested S693D as a phosphomimetic mutant may act as Drp1 phosphorylated by GSK3beta and resulted in this Drp1 mutant unable to be targeted to mitochondrial membrane (refers to Figure 9, the model). In contrast, S693A, is unable to be phosphorylated by GSKbeta and S693A may be targeted to mitochondria compared to S693D mutant, but lack of the function as a dominant negative mutant even though, unlike to K38A, it only has a partial deficiency in GTPase activity. The partial deficiency of GTPase activity of S693A may still compensate by Drp1 wild-type compared to S693D. However, the underlying molecular mechanism of S693A does not play a role interfering functions of Drp1 wild-type waits for further elucidation. Taken together, the GTPase activity of Drp1 is associated with and may be regulated by GSK3beta-mediated phosphorylation, which effects mitochondrial fission (causes elongated mitochondrial morphology) even though Drp1 assembly is not affected.

Tremendous progress has been achieved in elucidating the functional roles of phosphorylation on Drp1, providing significant evidence that mitochondria dynamics are regulated by phosphorylation and by various signaling cascades including CaMKIα, PKA, and Cdk1/Cyclin B [14]–[17], [19], [26]. The phosphorylation of Ser616 by Cdk1/Cyclin B enhances mitochondrial fission, resulting in proper distribution of mitochondria in mitosis. Whether this effect is correlated to Drp1 GTPase activity is unclear. Nevertheless, the phosphorylation of Ser637 by PKA has been proved to cause elongated mitochondria as well as decreased GTPase activity [14], [19]. In contrast, phosphorylation of Ser637 by CaMKIα, independent of the GTPase activity of Drp1, results in an increased affinity to Fis1 and causes mitochondrial fission in presence of Ca^2+^ influx [17]. We found that Ser693 phosphorylation by GSK3beta results in defective GTPase activity and elongated mitochondria (Figure 4 and 5). In comparison with previously reported phosphorylation sites, Ser616 and Ser637 of Drp1 [14]–[17], [19], using phosphomimetic mutants, the phenotype of Ser693 is more likely to be similar to Ser637, but is evidently different from Ser616 [19]. It is likely that Drp1 phosphorylation occurring at different sites in GED could result in different physiological consequences (Figure 5). Regarding the functional importance of the GED domain (region covering Ser616, Ser637, Ser693 sites), thus far the crystal structure of GED for dynamin oligomerization and stimulated GTP hydrolysis has been further proven to have functional implications which are suggested to be critical among dynamin family proteins [27].

It has been suggested that mitochondrial fragmentation is associated with apoptotic and non-apoptotic cell death [10], [20], [28], whereas inhibiting mitochondrial fission results in autophagy [20], [29]–[31]. We observed that ectopic expressed phosphomimetic mutant of Ser693, similar to another Drp1 dominant-negative mutant K38A and S637D, leads to an elongated mitochondrial phenotype and this finding prompted us to investigate the significance of the Ser693-phosphorylation-induced elongated mitochondria. Several recent studies indicated that mitochondrial fusion can serve a protective function, exchange mitochondrial DNA, reorganize mitochondrial cristae, and delay apoptosis [32], [33]. It is therefore reasonable that mitophagy, a particular form of selective mitochondrial autophagy, may result from alterations in mitochondrial morphology [20], [34]. Also, it has been proposed that mitochondria may also serve as docking sites for the formation of the autophagosomes in a process that depends on the tethering of the mitochondria to the endoplasmic reticulum [35]. Cribbs & Strack showed that Drp1 phosphorylation does result in an elongated mitochondria phenotype which protects against apoptotic insults [15]. Here we showed that mitochondria may acquire resistance to apoptotic events via down-regulating cytochrome c release, capase-3, -7 and PARP activations when elongated. Contradictorily, no inter-group difference was found for autophagy-related proteins, such as LC3B, p62, Atg5 and Beclin-1. This finding indicated that ectopic expression Drp1 mutants (K38A, S637D and S693D) caused elongated mitochondrial phenotype and resulted in acquired resistance to apoptosis rather than triggering autophagy (Figure 7 and 8). Notably, our results showed that S693D is the more effective group in protecting both non-neuronal and neuronal cells from apoptotic death compared to the S637D group (Figure 8). This phenomenon might have potential implications for detecting the Drp1 phosphorylation state as a biomarker in neurological diseases. For instance, do brain tumor victims retain anti-apoptosis-associated Drp1 mutants which are associated with tumorigenesis and/or equip tumor cells for resistance to cancer therapy [36] ? On the other hand, do patients with neurodegnerative disorders harbor unphosphorylated Drp1 mutants or S-nitroylation Drp1 mutants that are correlated to abnormality in mitochondrial fission [4], [37], [38]?

In support of the model in Figure 9 in which mitochondrial dynamics are regulated by phosphorylated Drp1, several lines of evidence, including the data presented here, indicate that phosphorylation on Ser637 or Ser693 of Drp1 diminishes mitochondrial fission, resulting in elongated mitochondria [14], [19]. Mitochondrial dynamics are regulated by the GTPase hydrolysis activity of proteins (Drp1, Opa1, Mfn1 and 2) resulting in mitochondrial fission or fusion [26]. Merrill et al. revealed that neurons were protected from diverse insults through remodeling by PKA/AKAP1 [39]. Since GSK3beta is ubiquitous in distribution in a cell, it might exhibit its function in many components through interaction with other proteins. Indeed, it has been reported that GSK3beta might be recruited to mitochondria through AKAP220 and interacts with PP1, 2, and 3 for dephosphorylation [40]. Mitochondrial morphological changes could also be involved in determining cell fate. Mitochondrial fission is involved in the initiation of apoptosis, whereas mitochondrial fusion is able to inhibit H_2_O_2_-induced mitochondrial fragmentation and ensuing apoptosis, but does not provoke protective autophagy. Here we demonstrate that Drp1 is phosphorylated by GSK3beta at Ser693 and by PKA at Ser637 leading mitochondria to undergo rapid elongation through the attenuation of GTPase activity, down-regulating cytochrome c release, capase-3, -7 and PARP activation (Figure 8). Interestingly, functionally expressed S637D caused mitochondrial elongation that cannot be inhibited by H89. This means inhibition of PKA signaling may only abolish the functions of endogenous Drp1 sequestering PKA phosphorylation at the Ser637 site, but not the phosphomimetic mutant. Nevertheless, inhibition of PKA-mediated phosphorylation at the Ser637 site, unlike the inhibition of GSK3beta-mediated phosphorylation at the Ser693 site, is insufficient to reverse the elongated mitochondria when comparing the results from the LiCl untreated- and LiCl-treated S693D groups. This result indicates that a kinase other than PKA may also be involved in phosphorylation at the Ser637 site causing an elongated mitochondria phenotype. In contrast, ectopic expressed S693D also leads to an elongated mitochondrial morphology and only a portion of the elongated mitochondria were reversed to a fragmented phenotype via inhibiting LiCl, a GSK3beta inhibitor. Either inhibition of GSK3beta or PKA signaling facilitates some morphological reversion of mitochondria from elongated to fragmented phenotype (Figure 6A & B). The underlying molecular mechanisms of incapability of inhibiting GSK3beta or PKA signaling to reverse mitochondrial morphology still wait for further clarification. Moreover, the exact roles corresponding to Drp1mutants, such as S616, S637, K679 and S693, in affecting the GED domain structure, GTPase activity, phosphorylating status, biological functions also needs to be validated in future studies.

In summary, we demonstrated directly that GSK3beta interacts with Drp1 and located Ser693 in the GED domain as a GSK3beta phosphorylation site. The required region of Drp1_634–690_ and K679 are crucial for GSK3beta interaction. We also revealed that mitochondrial elongation due to ectopic expressed S693D, S637D and K38A Drp1 mutants may be associated with enhanced resistance to H_2_O_2_-induced mitochondrial fragmentation and ensuing apoptosis via down-regulating cytochrome c release, capase-3, -7 and PARP activation, rather than inducing autophagy.

## Materials and Methods

### Cell culture and transfection

HeLa and HEK293 cells (ATCC) were cultured in DMEM (Gibco) supplemented with 10% fetal bovine serum (FBS), penicillin (100 U/mL) and streptomycin (100 µg/mL) at 37°C and 5% CO_2_. SH-SY5Y cells (ATCC), neuron-like cells, were cultured in D-MEM/F12 medium (Gibco) supplemented with 10% FBS, 1% nonessential amino acids (Gibco), 100 IU/ml penicillin, and 100 mg/ml streptomycin (Gibco) at 37°C in a humidified 5% CO_2_ incubator. Cells were passaged when they reached 80–90% confluence at 1: 5–6 with 0.05% trypsin. The Drp1 cDNA expression constructs have been described previously [5]. Cells were transfected for 24 hours in OptiMEM (Invitrogen) using Lipofectamine 2000 (Invitrogen). For western blots, 2×10^5^ cells were transfected using 2 µg DNA and 3 µL of Lipofectamine 2000. After 24 hours, cells were harvested in radioimmunoprecipitation assay (RIPA) buffer. For immunofluorescence, cells were transfected with 1 µg of DNA and 1.5 µl Lipofectamine 2000 and cultured for 24 hours. Thereafter, 5×10^4^ cells were seeded on coverslips and immunostained with fluorophore-conjugated antibodies. For H_2_O_2_ insult, HEK293, HeLa and SH-SY5Y cells were transfected with GFP alone, GFP-tagged Drp-1 wild-type and other mutants for 24 hours. Then cells were treated with 500 µM H_2_O_2_ for another 24 hours, then observed by confocal fluorescent microscopy or harvested for western blotting analysis. For inhibition of GSK3beta and PKA signaling, HeLa cells were transfected with GFP-tagged Drp1wt or other mutants for 24 hours. Then cells were treated with10 mM LiCl and 10 µM H89 for another 24 hours. Mitochondrial morphology of HeLa cells was observed by staining with Mitotracker under confocal microscopy. Cell nuclei were counter-stained by using 4′, 6-diamidino-2-phenylindole (DAPI, 1.5 µg/mL).

### Cloning, site-directed mutagenesis and DNA sequencing

To construct the pACT2-Drp1 plasmid for the yeast two-hybrid working assay, DNA fragments encoding Drp1 were amplified by PCR using high fidelity polymerase (Roche). The truncate 1–6 of Drp1 were amplified by PCR. These amplified fragments were digested by BamHI and XhoI, and they were also introduced into the BamHI and XhoI sites of the pACT2 or pAS2-1 vector (MATCHMAKER Two-Hybrid System 2, Clontech). Full-length Drp1 was inserted into the pEGFP-C1 vector using the BamHI and XhoI restriction sites. Full-length or truncated with or without point-mutated Drp1 fragments were also inserted into the pET-32a (+) vector using BamHI sites. Site-directed mutagenesis experiments to create the Drp1 mutants (K38A, S637D, S693A, and S693D) were carried out using the Quickchange system. All procedures were done according to the manufacturer's protocol (Stratagene) with minor modifications. The nucleotide sequencing was performed using a BigDye terminator v3.1 kit and the extended products were resolved on an ABI PRISM™ 3730 Genetic Analyzer (Applied Biosystems).

### Yeast two hybrid system

Standard techniques were used to carry out yeast two-hybrid screening [6]. Briefly, Drp1 and its variants were cloned in frame with the Gal4 DNA binding domain in the pAS2-1 vector and activated domain pACT2 to yield the bait plasmid and prey plasmid as indicated in the Figure 3. After 2–3 days transfection into the yeast, positive clones were able to grow on Trp, Leu, His dropout medium supplemented with 5 mM 3-aminotriazole (3-AT, an inhibitor of HIS3), and they turned blue in a beta-galactosidase filter assay. The power of protein-protein interaction was interpreted as described previously [5].

### Western blot analysis

Lysates were prepared using chilled cells on ice for 30 minutes in RIPA buffer, cleared by centrifugation. For detecting cytochrome C, we prepared cell lysates carefully avoiding destroy mitochondria using Mitochondria/Cytosol Fractionation Kit (Millipore) even though we did not prepare the mitochondrial/cytosolic fractions separately. Protein concentration was determined by the Bradford method and 20 µg per lane of lysate was resolved by SDS-polyacrylamide gel electrophoresis (PAGE) and transferred onto nitrocellulose membranes. Nonspecific binding was blocked by 1 hour incubation with blocking buffer before membranes were probed overnight at 4°C with primary antibodies (anti-cytochrome c, cleaved -caspase 3, cleaved-caspase 7, cleaved-PARP, -Bcl2, -LC3, -p62, -Beclin 1, -Atg5 and GFP antibody; beta-actin served as a protein loading control) diluted in blocking buffer (5% low-fat milk in Tris-buffered saline with 0.1% Tween-20 (TBS-T)). After extensive washing with TBS-T, specific bands were detected on Hyperfilm™ (GE Healthcare) using horseradish peroxidase (HRP)-conjugated secondary antibodies and the ECL detection system (GE Healthcare).

### In vitro kinase assay

The kinase reaction was carried out as described previously [21]. Briefly, the Drp1 variants were purified and incubated with GSK3beta (25 units, NEB) in kinase buffer [1 mM Na3VO4, 1 mM dithiothreitol, 2 mM EGTA, 25 mM Tris (pH 7.2), 10 mM MgCl_2_, 0.1 mM ATP, 0.5 mM PMSF, 10% glycerol, and 10 Ci of [γ-^32^P]ATP (GE Healthcare), 3000 Ci/mM]. The assays were carried out for 15 minutes at 30°C. The reaction was stopped by adding 2× sample buffer. Samples were denatured at 95°C for 5 min and separated by 8% SDS-PAGE. Signals were detected via autoradiography.

### GTPase activity assay

The cDNA of wild-type or mutant Drp1 was cloned into pET32A for 6His-Drp1 fusion protein expression. The soluble His-tagged wild-type or mutant Drp1 variants were purified from *Escherichia coli* BL21 (DE3) using Ni-Q affinity resin (QIAGEN). GTP hydrolysis by recombinant Drp1 was assayed using a simple, continuous, coupled GTP regenerating assay as described elsewhere [41] by measuring the depletion of NADH at OD.340 nm using a fluorescence reader (PowerWave XS2 (BioTek, USA)).

### Immunofluorescence and quantification of mitochondrial morphology

Cells were pre-incubated with 100 nM MitoTracker (Invitrogen) for 30 minutes, fixed in 3.7% paraformaldehyde (PFA) for 15 minutes and permeabilized with 0.1% Triton for 15 minutes at room temperature. Nonspecific binding was blocked for at least 30 minutes using blocking buffer (10% horse serum in PBS). Next, cells were incubated for 2 hours with primary antibodies in blocking buffer, washed 4 times with PBS, 5 minutes each, and incubated with FITC-anti-mouse IgG diluted 1∶300 in PBS. After washing again, the coverslips were mounted using Vectashield® (Vector Labs) containing DAPI (1.5 µg/mL), and wide-field epi-fluorescence images were acquired at room temperature with an Olympus FlowView confocal microscope system. Mitochondrial morphology was categorized by mitotracker staining in GFP-Drp1 cells expressing WT, K38A, S637D, K679A, S693A, or S693D as described in the study. Digital microscopic images of cells were acquired with a confocal microscope as described above.

### Statistics

Data from densitometry analysis and confocal fluorescent microscopy were indicated as continuous variables, and then subjected to testing for inter-group difference by paired T-test using PAWS Statistics version 18.0 software (SPSS, IBM). For multi-group comparisons, one-way ANOVA was used with Tukey's post-hoc test. A p-value less than 0.05 was viewed as statistically significant.

## Supporting Information

Figure S1 **The protein levels of ectopic expression of GFP-tagged Drp1 wt and mutants in HeLa cells.**
(TIF)

Figure S2 **Mitochondrial morphology of HeLa cells with Drp1 wt and K679A mutant.** HeLa cells were transfected with GFP-tagged Drp1 wt or K679A mutant for 24 hours. Mitochondrial morphology was observed by confocal fluorescent microscope with Mitotracker dye. Cell nuclei were counter-stained by DAPI. Insets are magnifications of the Mitotracker signal at the indicated areas. Inset 1 represents the non-transfected cells, and inset 2 indicates the transfected cells.
(TIF)

Figure S3 **Overexpressed Drp1 S693D can protect against H_2_O_2_-induced mitochondrial fragmentation and ensuing apoptosis but does not induce autophagy.** HEK293 cells were transfected with GFP alone, GFP-tagged Drp-1 wild-type and other mutants for 24 hours. Then cells were treated with 500 µM H_2_O_2_ for another 24 hours, and were lysed and detected by Western blotting using anti-cytochrome c, -caspase 3, -caspase 7, -PARP, -Bcl2, -LC3, -Beclin 1 and -Atg5 antibody, respectively. beta-actin served as a protein loading control. The data are representative of three independent experiments.
(TIF)
