# Supplementary figures and images for: GSK3beta-Mediated Drp1 Phosphorylation Induced Elongated Mitochondrial Morphology against Oxidative Stress
Source: PLoS One. 2012 Nov 20;7(11):e49112. doi: 10.1371/journal.pone.0049112 (PMC3502545; doi:10.1371/journal.pone.0049112)

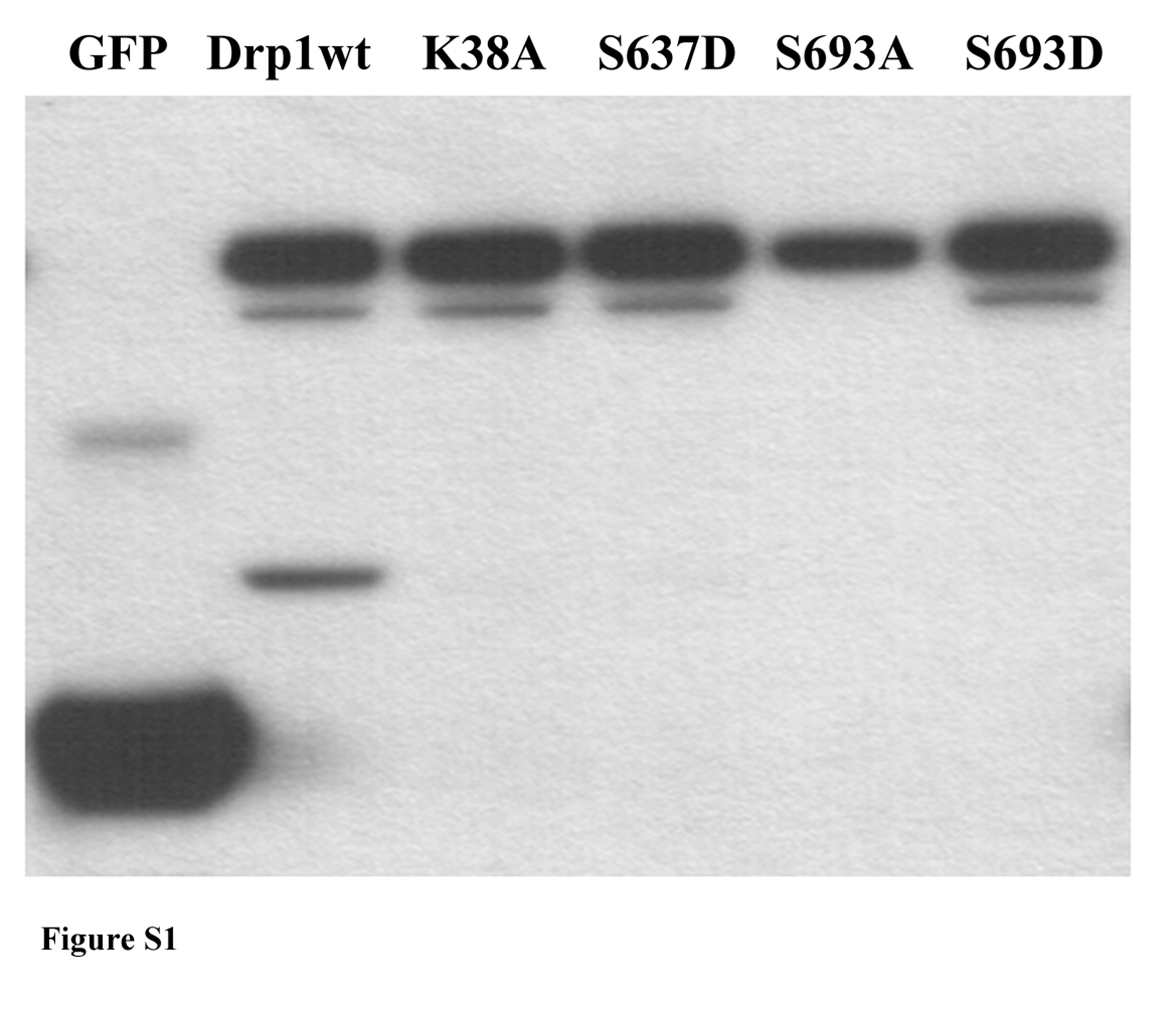

Supplement: Figure S1 — The protein levels of ectopic expression of GFP-tagged Drp1 wt and mutants in HeLa cells. (TIF) [file pone.0049112.s001.tif]

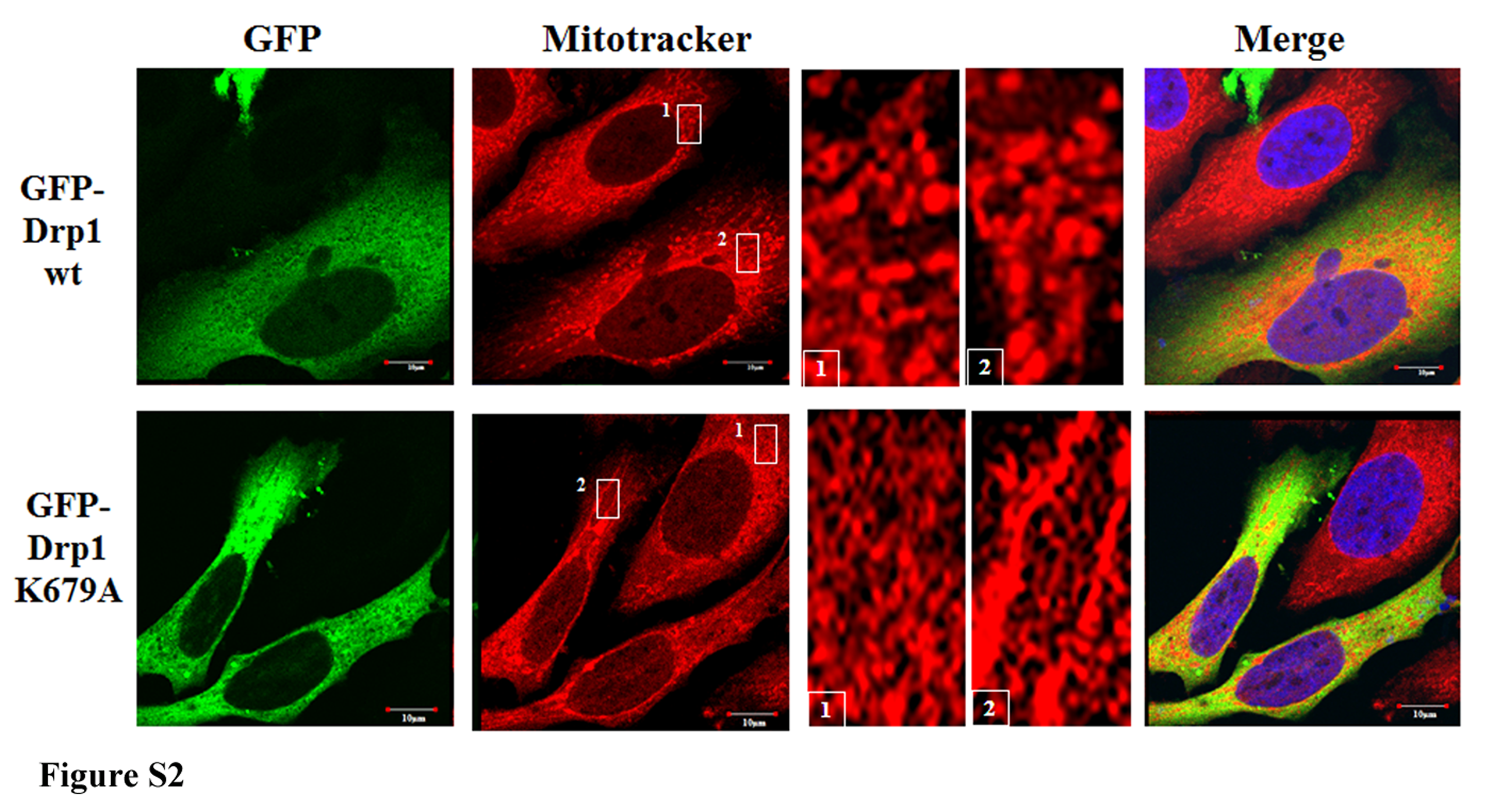

Supplement: Figure S2 — Mitochondrial morphology of HeLa cells with Drp1 wt and K679A mutant. HeLa cells were transfected with GFP-tagged Drp1 wt or K679A mutant for 24 hours. Mitochondrial morphology was observed by confocal fluorescent microscope with Mitotracker dye. Cell nuclei were counter-stained by DAPI. Insets are magnifications of the Mitotracker signal at the indicated areas. Inset 1 represents the non-transfected cells, and inset 2 indicates the transfected cells. (TIF) [file pone.0049112.s002.tif]

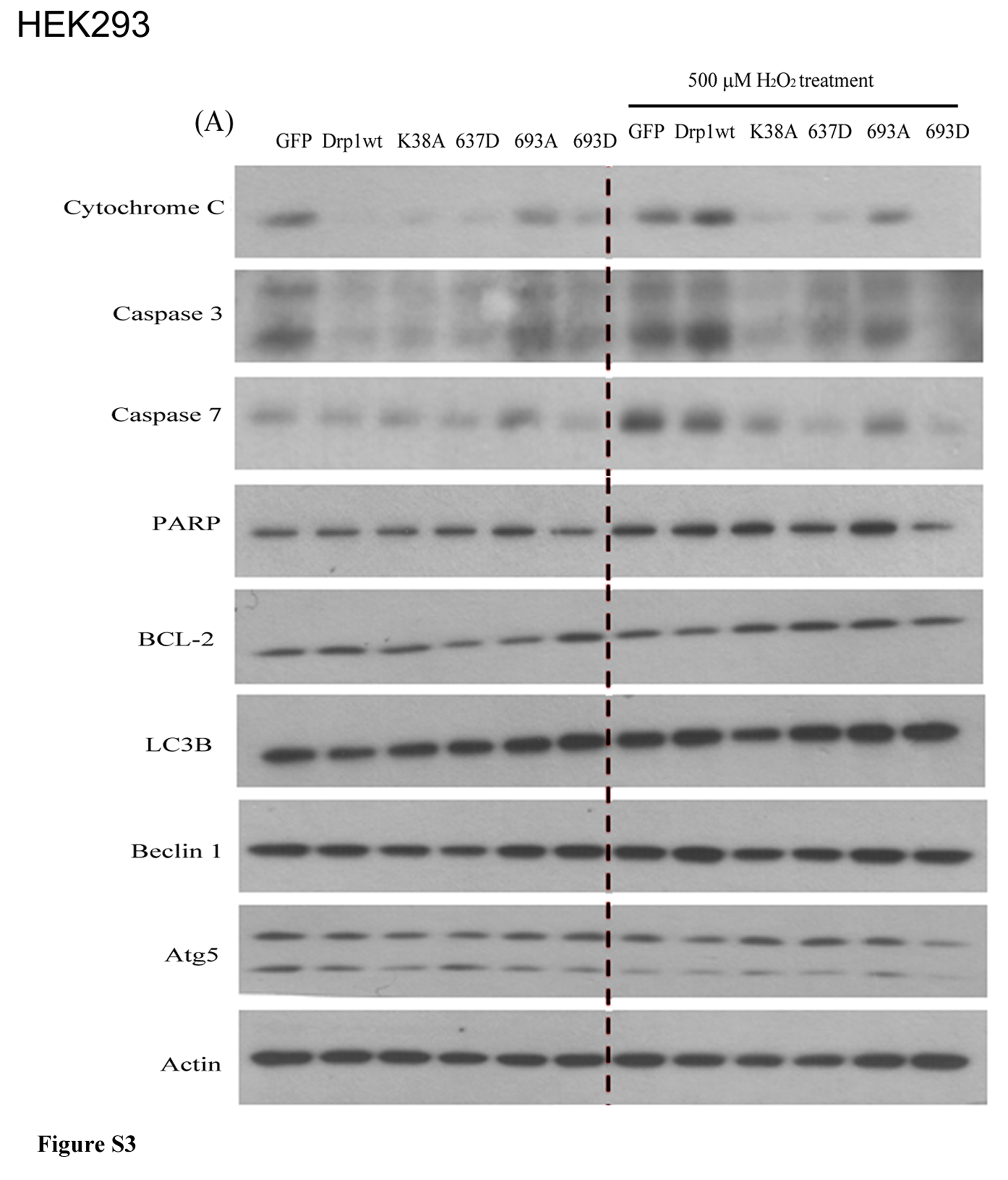

Supplement: Figure S3 — Overexpressed Drp1 S693D can protect against H2O2-induced mitochondrial fragmentation and ensuing apoptosis but does not induce autophagy. HEK293 cells were transfected with GFP alone, GFP-tagged Drp-1 wild-type and other mutants for 24 hours. Then cells were treated with 500 µM H2O2 for another 24 hours, and were lysed and detected by Western blotting using anti-cytochrome c, -caspase 3, -caspase 7, -PARP, -Bcl2, -LC3, -Beclin 1 and -Atg5 antibody, respectively. beta-actin served as a protein loading control. The data are representative of three independent experiments. (TIF) [file pone.0049112.s003.tif]
